# Intracranial Atherosclerosis Coexisting With White Matter Hyperintensities May Predict Unfavorable Functional Outcome in Patients With Acute Cerebral Ischemia

**DOI:** 10.3389/fneur.2020.609607

**Published:** 2020-12-21

**Authors:** Haiyan Liu, Yuehua Pu, Yilong Wang, Xinying Zou, Yuesong Pan, Changqing Zhang, Yannie O. Y. Soo, Thomas W. H. Leung, Xingquan Zhao, Ka Sing Lawrence Wong, Yongjun Wang, Liping Liu

**Affiliations:** ^1^Department of Neurology, Beijing Tiantan Hospital, Capital Medical University, Beijing, China; ^2^Department of Neurology, The Second Affiliated Hospital of Xuzhou Medical University, Xuzhou, China; ^3^China National Clinical Research Center for Neurological Diseases, Beijing, China; ^4^Center of Stroke, Beijing Institute for Brain Disorders, Beijing, China; ^5^Beijing Key Laboratory of Translational Medicine for Cerebrovascular Disease, Beijing, China; ^6^Department of Medicine and Therapeutics, Prince of Wales Hospital, Chinese University of Hong Kong, Hong Kong, China

**Keywords:** white matter hyperintensities, intracranial atherosclerosis (ICAS), stroke recurrence, small vessel disease (SVD), outcome

## Abstract

**Background and Purpose:** This study aimed to assess the effect of baseline white matter hyperintensities (WMH) on 1-year stroke recurrence and the functional outcome for patients with intracranial atherosclerosis (ICAS).

**Methods:** We analyzed 2,076 patients who were enrolled in the Chinese IntraCranial AtheroSclerosis (CICAS) study. ICAS and WMH were diagnosed by baseline magnetic resonance angiography. The primary outcomes were stroke recurrence and unfavorable functional outcome (modified Rankin Scale score 3–6) at 1 year.

**Results:** Of the 2,076 patients included in this study, 1,370 (65.99%) were men, and the mean age was 61.70 years. In total, 224 (10.79%) patients had no WMH and no ICAS, 922 (44.41%) patients had WMH and no ICAS, 157 (7.56%) patients had ICAS and no WMH, and 773 (37.24%) had both WMH and ICAS. During the follow-up period, 87 patients had a recurrent stroke and 333 had unfavorable outcomes at 1 year. Compared to WMH (–) ICAS (–) group, the adjusted odd ratios and 95% confidence interval for unfavorable functional outcome were 0.791 (0.470–1.332; *p* = 0.3779) in the WMH (+) ICAS (–) group, 1.920 (1.024–3.600; *p* = 0.0421) in the WMH (–) ICAS (+) group, and 2.046 (1.230–3.403; *p* = 0.0058) in the WMH (+) ICAS (+) group. There was no significant difference in stroke recurrence risk among the four groups.

**Conclusion:** ICAS coexisting with WMH may predict an unfavorable functional outcome at 1 year, but not stroke recurrence.

## Introduction

Intracranial atherosclerosis (ICAS) is likely to be the most common stroke subtype worldwide (1). It accounts for about 15% of Caucasian patients with ischemic attack or stroke (2) and nearly 50% of ischemic strokes in Asia (1, 3, 4). Cerebral small vessel disease (SVD) is another kind of common cerebrovascular disease, which manifests as recent small subcortical infarcts, lacunes, white matter hyperintensities (WMH), perivascular spaces, microbleeds, and brain atrophy on neuroimaging (5). SVD may also have racial differences, and an observational study found that Han Chinese had a higher prevalence of confluent WMH than white Australians, but had a similar prevalence of lacunes and microbleeds (6). Some studies have suggested that patients with ICAS may be particularly prone to having coexistent SVD (7–9). Patients with multiple ICAS lesions, occlusive lesions, and atherosclerotic lesions in the posterior circulation were more likely to coexist with WMH (10). Previous studies have found that WMHs were associated with risk of incident stroke, ischemic stroke, intracerebral hemorrhage, dementia, Alzheimer's Disease, and death (11). A study from the PICASSO (Prevention of Cardiovascular Events in Ischemic Stroke Patients with High Risk of Cerebral Hemorrhage) trial showed that the severity of WMH on baseline brain magnetic resonance imaging scans may be associated with a 2.15-fold risk of stroke, 2.11-fold risk of ischemic stroke, and 3.72-fold risk of hemorrhagic stroke (12).

It is still uncertain whether the presence of SVD on baseline magnetic resonance imaging could affect the stroke recurrent risk and functional outcome of patients with ICAS. A subgroup analysis of the Stenting and Aggressive Medical Management for Preventing Recurrent Stroke in Intracranial Stenosis (SAMMPRIS) showed that the SVD image markers are not independently associated with an increased risk of stroke in patients with ICAS (13). Lau et al. validated a total small vessel disease score in two independent prospective studies and found that a higher score was associated with an increased risk of recurrent ischemic stroke and intracerebral hemorrhage (14). A study from the Clopidogrel in High-Risk Patients with Acute Non-disabling Cerebrovascular Events (CHANCE) trial, indicated that SVD and ICAS may have different levels of risk for future strokes. SVD was associated with more disability and bleeding events, and ICAS was associated with an increased risk of stroke and disability in patients with minor stroke and TIA at 3 months (15).

This study hypothesized that ICAS and WMH may interact with each other, resulting in an increased risk of unfavorable functional outcome and stroke recurrence. The study aimed to assess the effect of baseline WMH on 1-year stroke recurrence and the functional outcome for patients with acute cerebral ischemia in the Chinese Intracranial Atherosclerosis (CICAS) study database.

## Materials and Methods

### Subjects

From October 2007 to June 2009, a total of 2,864 patients were recruited into the CICAS study (4). We excluded 444 patients who did not have interpretable images for the presence of white matter changes and 344 patients with extracranial large artery stenosis or occlusion. Finally, 2,076 patients were included in this study. The CICAS study was a multicenter, hospital-based cohort study that included 22 hospitals in mainland China and the Hong Kong Special Administrative Region. The study was approved by the Institutional Review Boards of the participating hospitals. Details of the CICAS study design and the definition of baseline characteristics have been published previously (4). This study recruited patients with cerebral ischemia, aged from 18 to 80, admitted within 7 days of symptom onset. We excluded patients who were clinically unstable and those that required close monitoring or were disabled before admission, physically or subjectively unable to comply with magnetic resonance (MR) examination or had severe comorbidity, and those who were presumed to have had a cardioembolic stroke such as atrial fibrillation.

### Brain MRI Assessment

All MR images were stored in digital format and were read by two readers blinded to the clinical information of subjects (4). Intracranial stenosis or occlusion was estimated by 3-dimensional time-of-flight MR angiography (3D TOF MRA). ICAS was defined as stenosis more than or equal to 50% on MRA for the main intracranial arteries. Intracranial arterial segments included the distal internal carotid artery (ICA), middle cerebral artery (MCA) (M1 and M2 segment), anterior cerebral artery (ACA) (A1 and A2 segment), posterior cerebral artery (PCA) (P1 and P2 segment), and basilar artery (BA). Duplex color Doppler ultrasound or contrast-enhanced MRA were used for extracranial carotid vessels. WMH was defined as a hyperintense lesion on both T2-weighted imaging and FLAIR but was usually not seen on T1-weighted imaging or showed faint hypointensity (5). DWI was used to differentiate acute ischemic stroke lesions from WMH. The severity of WMH was assessed according to the Fazekas scale (16). Scores in periventricular white matter hyperintensities (PWMH) and deep white matter hyperintensities (DWMH) were evaluated separately and summed together as Fazekas scores. The total Fazekas score was classified into two categories: 0–3 and 4–6. According to the presence of ICAS or WMH, the patients were classified into four groups: WMH (–) ICAS (–), WMH (+) ICAS (–), WMH (–) ICAS (+), and WMH (+) ICAS (+). WMH (–) was defined as a Fazekas score equal to 0.

### Follow-Up Assessment

We monitored the included patients for 1 year through telephone or face to face consultations with trained research personnel from the follow-up center of the Beijing Tiantan Hospital and the Hong Kong Prince of Wales Hospital. The primary outcomes were stroke recurrence and unfavorable functional outcome (modified Rankin Scale score 3–6). Stroke recurrence was defined as sudden functional deterioration in neurological status with a decrease in the National Institutes of Health Stroke Scale (NIHSS) score of four or more, or a new focal neurological deficit of vascular origin lasting >24 h, including recurrent ischemic or hemorrhagic stroke (4).

### Statistical Methods

All statistical analyses were performed using SAS software (version 9.4; SAS Institute, Cary, NC, USA). Two-sided *P*-values <0.05 were considered statistically significant. Continuous variables were summarized as median (interquartile range) or mean (SD). Categorical variables were presented as numbers (percentages). The χ^2^ test (or Fisher exact test, when appropriate) was used to test differences in proportions for categorical variables. A Wilcoxon signed rank test was used to test differences in median for the continuous variables. The cumulative probabilities of stroke recurrence over time were estimated by the Kaplan–Meier product-limit method and were compared among WMH (–) ICAS (–), WMH (+) ICAS (–), WMH (–) ICAS (+), and WMH (+) ICAS (+) group using the log-rank test. Cox proportional hazards regression analyses were used to estimate the hazards ratio of each group for stroke recurrence adjusted by potential confounders. For the unfavorable functional outcome at 1 year, odds ratio (OR), and 95% confidence interval (CI) were given and logistic regression was used for adjusting confounders. All tests were two-sided with a significance level fixed at 5%.

## Results

Among the 2,076 patients included in this study, 1,370 (65.99%) were men, and the mean (SD) of age was 61.70 (11.32) years. Two hundred and twenty-four (10.79%) patients had no WMH and no ICAS, 922 (44.41%) patients had WMH and no ICAS, 157 (7.56%) patients had ICAS and no WMH, and 773 (37.24%) had both WMH and ICAS. The baseline characteristics for each group are shown in Table 1. Patients with both WMH and ICAS were older and more likely to have diabetes, hypertension, hyperhomocysteinemia, and a history of stroke. The median (interquartile range) of baseline Fazekas score in the WMH (+) group was 3 (2–4).

**Table 1:** Baseline characteristics of participants.

**Characteristics**	**WMH (–) ICAS (–) (*n* = 224)**	**WMH (+) ICAS (–)(*n* = 922)**	**WMH (–) ICAS (+) (*n* = 157)**	**WMH (+) ICAS (+)(*n* = 773)**	***P*-value**
Male sex, *n* (%)	151 (67.41)	611 (66.27)	120 (76.43)	488 (63.13)	0.0136
Age, mean (SD), y	53.73 (10.71)	63.09 (10.53)	52.33 (11.74)	64.25 (10.28)	<0.0001
BMI, mean (SD), kg/m^2^	24.82 (3.43)	24.53 (3.09)	25.08 (3.29)	24.37 (3.03)	0.2333
Diabetes, *n* (%)	51 (22.77)	301 (32.65)	55 (35.03)	315 (40.75)	<0.0001
Hypertension, *n* (%)	132 (58.93)	734 (79.61)	92 (58.60)	654 (84.61)	<0.0001
Hyperlipidemia, *n* (%)	170 (75.89)	689 (74.73)	123 (78.34)	582 (75.29)	0.8047
Hyperhomocystinemia, *n* (%)	48 (21.43)	189 (20.50)	38 (24.20)	233 (30.14)	<0.0001
Family history of stroke, *n* (%)	26 (11.61)	72 (7.81)	25 (15.92)	73 (9.44)	0.0080
Current smoker, *n* (%)	82 (36.61)	308 (33.41)	82 (52.23)	226 (29.24)	<0.0001
Heavy drinker, *n* (%)	12 (5.36)	37 (4.01)	15 (9.55)	29 (3.75)	0.0103
History of stroke, *n* (%)	25 (11.16)	219 (23.75)	22 (14.01)	255 (32.99)	<0.0001
Heart disease, *n* (%)	7 (3.13)	79 (8.57)	9 (5.73)	66 (8.54)	0.0277
Peripheral vascular disease, *n* (%)	1 (0.45)	7 (0.76)	1 (0.64)	4 (0.52)	0.9132
NIHSS score at admission, median (IQR)	2 (0–5.5)	3 (2–5)	4.5 (2–9)	4 (2–8)	<0.0001
Pattern of infarct, *n* (%)					<0.0001
No infarct	83 (37.73)	245 (27.01)	23 (14.65)	128 (16.67)	
Cortical infarct	8 (3.64)	20 (2.21)	7 (4.46)	42 (5.47)	
Subcortical infarct	69 (31.36)	439 (48.40)	43 (27.39)	289 (37.63)	
Cortical and subcortical	18 (8.18)	39 (4.30)	60 (38.22)	141 (18.36)	
Infratentorial	41 (18.64)	160 (17.64)	22 (14.01)	149 (19.40)	
Supratentorial and infratentorial	1 (0.45)	4 (0.44)	2 (1.27)	19 (2.47)	
Number of acute infarcts, *n* (%)					<0.0001
No acute infarct	83 (37.73)	245 (27.01)	23 (14.65)	127 (16.54)	
Single infarct	125 (56.82)	621 (68.47)	97 (61.78)	530 (69.01)	
Multiple infarct	12 (5.45)	41 (4.52)	37 (23.57)	111 (14.45)	
In-hospital treatment, *n* (%)					
Thrombolysis therapy	11 (4.91)	24 (2.60)	12 (7.64)	19 (2.46)	0.0021
Antithrombotic therapy	215 (95.98)	879 (95.34)	153 (97.45)	755 (97.67)	0.0634
Dual Antiplatelet	21 (9.38)	62 (6.72)	25 (15.92)	92 (11.90)	0.0001
Statins	164 (73.21)	684 (74.19)	121 (77.07)	602 (77.88)	0.2560
Blood pressure-lowering therapy	89 (39.73)	550 (59.65)	54 (34.39)	406 (52.52)	<0.0001

*ICAS, intracranial atherosclerosis; WMH, white matter hyperintensities; BMI, Body Mass Index; IQR, interquartile range; NIHSS, National Institutes of Health Stroke Scale; SD, Standard Deviation*.

**Figure 1:**
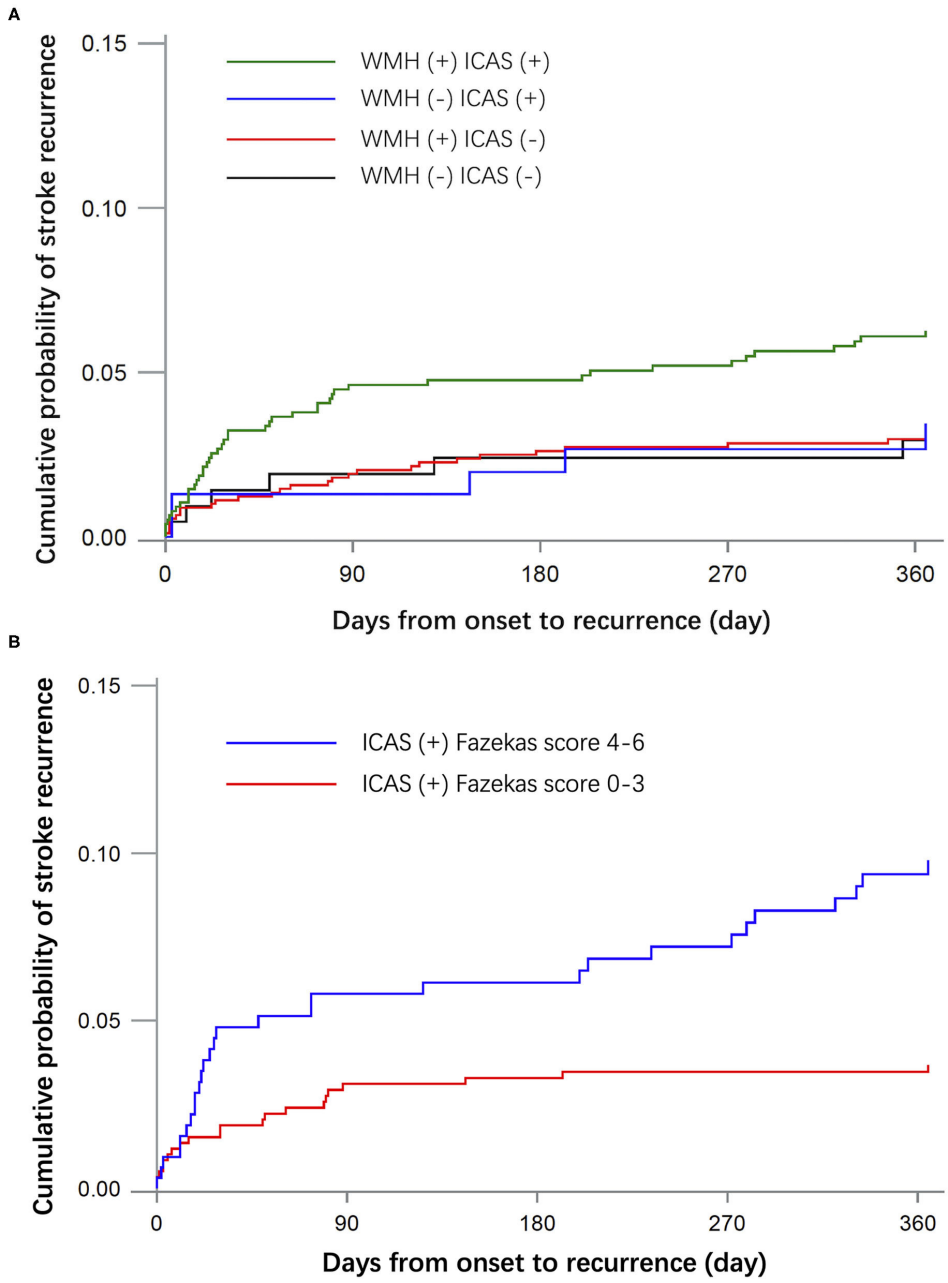
Kaplan-Meier curves of recurrent stroke within 1 year. **(A)** Shows the cumulative incidence of recurrent stroke in groups WMH (–) ICAS (–), WMH (+) ICAS (–), WMH (–) ICAS (+) and WMH (+) ICAS (+). **(B)** Shows the cumulative incidence of recurrent stroke in patients with ICAS by different severity of WMH.

During the follow-up period, 87 patients had a recurrent stroke and 333 had unfavorable outcomes at 1 year. The predictors of recurrent stroke in 2,076 patients are shown in Table 2. Patients with recurrent stroke were older and a higher percentage had hypertension, history of stroke, heart disease, multiple infarction, and ICAS. The stroke recurrence rate was 6 (2.68%) in the WMH (–) ICAS (–) group, 30 (3.25%) in the WMH (+) ICAS (–) group, 5 (3.18%) in the WMH (–) ICAS (+) group, 46 (5.95%) in the WMH (+) ICAS (+) group. Compared to the WMH (–) ICAS (–) group, the hazard ratio (HR) and 95% confidence interval (CI) was 0.682 (0.270–1.721), 1.124 (0.340–3.719), and 1.263 (0.510–3.131) in each group, respectively, after adjustment by age, sex, diabetes, hypertension, hyperhomocysteinemia, family history of stroke, current smoker, heavy drink, history of stroke, and heart disease. There was no significant difference between groups for recurrent risk of stroke at 1 year. For patients with ICAS, the stroke recurrent rate had no significant difference between Fazekas score 0–3 and 4–6. The HR (95%CI) of stroke recurrence in the patients in each group are shown in Table 3. Kaplan-Meier curves of recurrent stroke showed in Figure 1.

**Table 2:** Predictors of recurrent stroke in 2,076 patients (Univariate analysis).

**Characteristics**	**No recurrent stroke (*n* = 1989)**	**Recurrent stroke(*n* = 87)**	***P-*value**
Male sex, *n* (%)	1,317 (66.21)	53 (60.92)	0.3075
Age, mean (SD), y	61.55 (11.29)	65.20 (11.39)	0.0027
BMI, mean (SD), kg/m^2^	24.53 (3.11)	24.82 (3.59)	0.8559
Diabetes, *n* (%)	685 (34.44)	37 (42.53)	0.1210
Hypertension, *n* (%)	1,535 (77.17)	77 (88.51)	0.0130
Hyperlipidemia, *n* (%)	1,495 (75.16)	69 (79.31)	0.3798
Hyperhomocystinemia, *n* (%)	490 (24.64)	18 (20.69)	0.4021
Family history of stroke, *n* (%)	184 (9.25)	12 (13.79)	0.1561
Current smoker, *n* (%)	682 (34.29)	16 (18.39)	0.0021
Heavy drinker, *n* (%)	92 (4.63)	1 (1.15)	0.1250
History of stroke, *n* (%)	484 (24.33)	37 (42.53)	0.0001
Heart disease, *n* (%)	147 (7.39)	14 (16.09)	0.0030
Peripheral vascular disease, *n* (%)	13 (0.65)	0	1.0000^*^
NIHSS score at admission, median (IQR)	3 (1–6)	5 (3–10)	<0.0001
Pattern of infarct, *n* (%)			0.0649
No infarct	469 (23.87)	10 (11.49)	
Cortical infarct	74 (3.77)	3 (3.45)	
Subcortical infarct	804 (40.92)	36 (41.38)	
Cortical and subcortical	245 (12.47)	13 (14.94)	
Infratentorial	348 (17.71)	24 (27.59)	
Supratentorial and infratentorial	25 (1.27)	1 (1.15)	
Number of acute infarcts, *n* (%)			0.0044
No acute infarct	468 (23.82)	10 (11.49)	
Single infarct	1,311 (66.72)	62 (71.26)	
Multiple infarct	186 (9.47)	15 (17.24)	
ICAS, *n* (%)	879 (44.19)	51 (58.62)	0.0081
Fazekas score, median (IQR)	2 (1–4)	3 (2–4)	0.0008
In-hospital treatment, *n* (%)			
Thrombolysis therapy	60 (3.02)	6 (6.90)	0.0435
Antithrombotic therapy	1,925 (96.78)	77 (88.51)	<0.0001
Dual Antiplatelet	186 (9.35)	14 (16.09)	0.0370
Statins	1,503 (75.57)	68 (78.16)	0.5808
Blood pressure-lowering therapy	1,052 (52.89)	47 (54.02)	0.8360

* *p-value was calculated by Fisher's exact test; ICAS, intracranial atherosclerosis; WMH, white matter hyperintensities; BMI, Body Mass Index; IQR, interquartile range; NIHSS, National Institutes of Health Stroke Scale; SD, Standard Deviation*.

**Table 3:** Recurrent stroke in patients with WMH or ICAS.

	**Stroke recurrence, *n* (%)**	**HR (95%CI)**	***P-*value**	Adj. HR (95%CI)^*^	***P-*value**
WMH (–) ICAS (–)	6 (2.68)	1 (Reference)		1 (Reference)	
WMH (+) ICAS (–)	30 (3.25)	1.058 (0.437–2.563)	0.9004	0.682 (0.270–1.721)	0.4176
WMH (–) ICAS (+)	5 (3.18)	1.146 (0.350–3.755)	0.8216	1.124 (0.340–3.719)	0.8477
WMH (+) ICAS (+)	46 (5.95)	2.171 (0.926–5.089)	0.0745	1.263 (0.510–3.131)	0.6140
ICAS (+) Fazekas score 0–3	21 (3.48)	1 (Reference)		1 (Reference)	
ICAS (+) Fazekas score 4–6	30 (9.20)	2.658 (1.516–4.661)	0.0006	1.850 (0.977–3.505)	0.0591

* *The number of patients included was 2,061. Adjusted by age, sex, diabetes, hypertension, hyperhomocysteinemia, family history of stroke, current smoker, heavy drink, history of stroke, heart disease*.

*ICAS, intracranial atherosclerosis; WMH, white matter hyperintensities; CI, confidence interval; HR, hazard ratio*.

**Figure 2:**
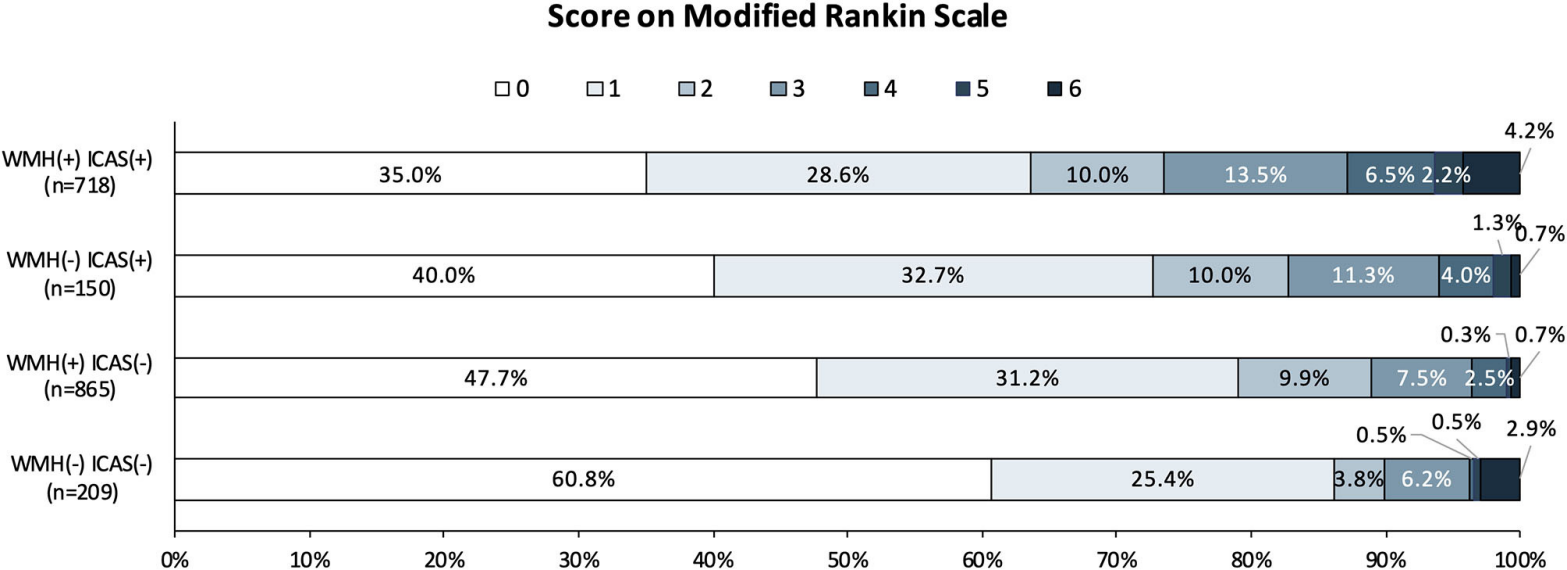
Distribution of mRS score at 1 year in the four groups. Scores on the modified Rankin scale range from 0 to 6, with 0 indicating no symptoms; 1, no clinically significant disability; 2, slight disability (able to handle own affairs without assistance but unable to carry out all previous activities); 3, moderate disability requiring some help, but able to walk unassisted; 4, moderately severe disability (unable to attend body needs and unable to walk); 5, severe disability (requiring constant nursing care and attention); and 6, death. mRS scores at 1 year were missing in 134 cases.

For unfavorable functional outcome at 1 year, there were 21 (10.05%) patients in the WMH (–) ICAS (–) group, 96 (28.83%) in the WMH (+) ICAS (–) group, 26 (7.81%) in the WMH (–) ICAS (+) group, and 190 (26.46%) in the WMH (+) ICAS (+) group. Compared to the WMH (–) ICAS (–) group, the adjusted odd ratios (OR) and 95% CI were 0.791 (0.470–1.332), 1.920 (1.024–3.600), 2.046 (1.230–3.403) in the other three groups, respectively. The OR (95%CI) of unfavorable outcomes in patients from each group are shown in Table 4. The distribution of Functional Scores at 1 year are shown in Figure 2.

**Table 4:** Unfavorable functional outcome at 1 year in patients with WMH or ICAS.

	**mRS 3–6, *n* (%)**	**OR (95%CI)**	***P*-value**	Adj. OR (95%CI)^*^	***P*-value**
WMH (–) ICAS (–)	21 (10.05)	1 (Reference)		1 (Reference)	
WMH (+) ICAS (–)	96 (28.83)	1.118 (0.679–1.840)	0.6620	0.791 (0.470–1.332)	0.3779
WMH (–) ICAS (+)	26 (7.81)	1.877 (1.012–3.483)	0.0459	1.920 (1.024–3.600)	0.0421
WMH (+) ICAS (+)	190 (26.46)	3.221 (1.992–5.209)	<.0001	2.046 (1.230–3.403)	0.0058
ICAS (+) Fazekas score 0–3	132 (23.04)	1 (Reference)		1 (Reference)	
ICAS (+) Fazekas score 4–6	84 (28.47)	1.330 (0.967–1.830)	0.0797	1.006 (0.701–1.445)	0.9732

* *The number of patients included was 1,942. Adjusted by age, sex, diabetes, hypertension, hyperhomocysteinemia, family history of stroke, current smoker, heavy drink, history of stroke, heart disease*. *ICAS, intracranial atherosclerosis; WMH, white matter hyperintensities; CI, confidence interval; OR, odds ratio*.

## Discussion

The findings of this study are consistent with those of other recently reported trials (13, 15), which indicated that the presence of WMH on baseline magnetic resonance imaging is not independently associated with an increased risk of stroke recurrence in patients with ICAS. The coexistence of intracranial atherosclerosis with changes in white matter may predict an unfavorable functional outcome at 1 year. Although the 1-year stroke recurrence rate in the ICAS and WMH group was higher than that of other groups, there was no significant difference after adjusting by confounders, suggesting that age, baseline NIHSS score, the number of acute infarctions, and medical history may be more relevant in stroke recurrence than WMH alone. For patients with ICAS, the severity of WMH had no significant correlation with the risk of stroke recurrence and functional outcome.

WMH is a common imaging feature that is associated with small-vessel disease (SVD) and related to stroke incidence, dementia, or death (11). SVD is common in patients with ICAS. Kwon et al. (13) observed that nearly half of ICAS patients had SVD on a baseline MRI. Lee et al. (9) reported a significant association between WMH and stroke subtypes. The large-artery-disease group in this study had a higher prevalence of WMH than other groups (55.4% in the large-artery-disease group, 30.3% in the lacunar group, and 14.3% in the cardioembolic). In a CICAS study, 41.45% of acute ischemic stroke or TIA patients had WMH at baseline MRI and patients with ICAS had a higher percentage of WMH than those without (45.77% vs. 37.67%) (4). However, there are limited data on the effect of WMH on stroke recurrence or functional outcome in patients with acute cerebral ischemia. A study from the SAMMPRIS trial showed that the presence of SVD on baseline magnetic resonance imaging was not independently associated with an increased risk of stroke in patients with ICAS (13). Data from the CHANCE trial found that SVD was associated with more disability and bleeding events and that ICAS is associated with an increased risk of stroke and disability in patients with minor stroke and TIA at 3 months, which implies that SVD and ICAS may represent different vascular pathologies and play distinct roles in stroke outcomes (15).

How to explain the results by the pathogenesis? The mechanisms of ICAS-related stroke include parent artery atherosclerosis occluding penetrating artery, artery to artery embolism, hypoperfusion, and mixed mechanisms). Different pathogenesis has different recurrence risk. Previous reports showed that ICAS with multiple infarctions (indicated by an artery to artery mechanism) (17), borderzone infarcts and impaired collateral flow (hemodynamic markers) (18), or a mixed mechanism of artery to artery embolism and hypoperfusion (19) were more likely to have recurrent stroke. WMHs are often considered to be a consequence of chronic hypoperfusion (20). Patients with hemodynamically more severe ICAS are more likely to have more severe ipsilateral WMH (21). Some researchers believe that impaired cerebral blood flow is one of the physiopathology mechanisms of WMH (22). However, the relationship between WMH and ICAS is still not clear. A meta-analysis study reviewed available published (and unpublished) research on cerebral blood flow (CBF) in small vessel disease, and data showed that a high WMH load is associated with lower CBF, but they are not causally related. In cross-sectional studies, low CBF was observed in most patients with more WMHs. The association was less pronounced after removing non-age matched subjects and those with dementia, which suggests that the underlying association is between reduced CBF and age or dementia rather than just WMH (20). Therefore, ICAS and WMH represent different types of pathophysiology. The ICAS-related recurrence of stroke correlates with plaque stability, hemodynamics, and collateral circulation, while the mechanisms of stroke recurrence related to WMH are more complex and involve arteriolar tortuosity, reduced vessel density, and occlusive venous collagenosis (23). WMH is usually associated with brain dysfunction, and ICAS is associated with stroke recurrence.

This study had several limitations. First, the included data was, in some cases, older, which could cause bias due to the advancement of medical treatment strategies such as dual anti platelet and high intensity statin. This was also a hospital-based study involving upper first-class hospitals, and most of the enrolled patients had a minor stroke, meaning that selective bias affects the included population. Second, the study included patients within seven days of onset, the rate of stroke recurrence might have been underestimated. Third, we did not distinguish the subtypes of recurrent stroke. Finally, other manifestations of cerebral small vessel disease (recent small subcortical infarcts, lacunes, perivascular spaces, microbleeds, and brain atrophy on neuroimaging) were not analyzed in this study.

In conclusion, the presence of WMH on baseline magnetic resonance imaging is not an independent predictor of stroke recurrence for patients with acute cerebral ischemia. The co-existence of intracranial atherosclerosis with changes in white matter may predict an unfavorable functional outcome at 1 year. For patients with ICAS, the severity of WMH had no significant correlation with the risk of stroke recurrence and functional outcome. The manifestations of WMH with different pathophysiological mechanisms may be different in images and the visual features of ICAS-related WMH need to be further explored in future studies.

## Data Availability Statement

Requests for access to the data reported in this paper will be considered by the corresponding author.

## Ethics Statement

The studies involving human participants were reviewed and approved by Beijing Tiantan Hospital of Capital Medical University Institutional Review Board. The patients/participants provided their written informed consent to participate in this study.

## Author Contributions

HL and YPu undertook the literature search, data analysis and interpretation, figures, and wrote the manuscript. LL, XZh, YiW, YoW, TL, and KW contributed to the study design, data analysis, data interpretation, and provided comments on the manuscript. XZo and YS contributed to data collection and image analysis and interpretation. CZ contributed to the image interpretation of white matter lesions. YPa contributed to data analysis and interpretation and figures. All authors contributed to the article and approved the submitted version.

## Conflict of Interest

The authors declare that the research was conducted in the absence of any commercial or financial relationships that could be construed as a potential conflict of interest.
